# Engagement, Acceptability, and Impact of a Parenting App to Support Infant Sleep and Parent Well-Being: Quasi-Experimental Study

**DOI:** 10.2196/88948

**Published:** 2026-06-11

**Authors:** Jessica Appleton, Anne-Marie Maxwell, Heilok Cheng, Sarah Taki, Deborah Stockton, Judith Fethney, Ann Debelin, Elizabeth Denney-Wilson, Nicole Reilly

**Affiliations:** 1 Tresillian Family Care Centres, Belmore, Sydney, NSW, Australia; 2 Sydney Institute for Women, Children and their Families, Sydney Local Health District, Sydney, NSW, Australia; 3 School of Nursing and Midwifery, Faculty of Health, University of Technology Sydney, 235 Jones Street, Ultimo, NSW, 2007, Australia, 61 0295144812; 4 The Centre of Research Excellence in Translating Early Prevention of Obesity in Childhood (EPOCH-Translate CRE), Faculty of Medicine and Health, University of Sydney, Sydney, NSW, Australia; 5 Susan Wakil School of Nursing and Midwifery, Faculty of Medicine and Health, The University of Sydney, Sydney, NSW, Australia; 6 School of Psychological Sciences, Faculty of Medicine, Health, and Human Sciences, Macquarie University, Sydney, NSW, Australia; 7 Prevention Research Collaboration, Faculty of Medicine and Health, The University of Sydney, Sydney, NSW, Australia; 8 Population Health Research & Evaluation Hub, Population Health Department, Sydney Local Health District, Sydney, NSW, Australia; 9 School of Public Health, Faculty of Health, University of Technology Sydney, Sydney, NSW, Australia; 10 College of Human and Social Futures, School of Education, University of Newcastle Australia, Sydney, NSW, Australia; 11 Graduate School of Medicine, Faculty of Science, Medicine and Health, University of Wollongong, Wollongong, NSW, Australia; 12 Discipline of Psychiatry and Mental Health, School of Clinical Medicine, University of New South Wales and Perinatal & Women’s Mental Health Unit, St John of God Burwood Hospital, Sydney, NSW, Australia

**Keywords:** mobile applications, mobile health, mHealth, digital health, smartphone, infant, parenting, self-efficacy, sleep, fatigue, anxiety, depression, perinatal mental health

## Abstract

**Background:**

Many parents today use digital or mobile health (mHealth) resources for parenting information and support. Thus, interventions or programs to support parenting delivered in this way are a rapidly growing area of innovation and research. Evidence indicates that face-to-face interventions that provide education and support for parents about infant sleep can have positive impacts on both infant sleep and maternal mood. However, there is limited research on the delivery of such interventions via digital or mHealth platforms. SleepWellBaby is an infant sleep mobile app for parents with infants and young children. The app provides parents with a 7-day program that may be repeated according to individual user demand, with information and interactive features aimed at supporting parents to understand infant sleep, and providing advice about strategies to support sleep in a responsive way.

**Objective:**

The study objectives were (1) to determine engagement with, and acceptability of, an mHealth-delivered parenting program to support infant sleep and (2) to investigate the impact of the program on parental fatigue, emotional health, and parenting competence, confidence, and self-efficacy.

**Methods:**

A multimethod quasi-experimental pre-post study design was used. Participants were parents of infants aged under 6 months, recruited through online advertising. Participants completed baseline questionnaires, then 2 further follow-up questionnaires at 7 days and 30 days post baseline. These questionnaires captured participant demographics (baseline) and perceptions of the program (follow-up), in addition to measuring parental fatigue (Fatigue Severity Scale), parent sense of competence, confidence, and self-efficacy (Me as a Parent Scale), and symptoms of anxiety (Generalized Anxiety Disorder 7-item scale) and depression (Edinburgh Postnatal Depression Scale) at all time points. Open-ended text responses were also collected at the 2 follow-up surveys. Questionnaire data were linked with participants’ engagement with the program (Engagement Index), measured using app analytics.

**Results:**

Participants’ (baseline: n=700; 7-d follow-up: n=339; and 30-d follow-up: n=220) engagement with the program varied; however, the majority reported cessation of app use by 1 month. Most participants were broadly satisfied with the program, finding the app easy to use and understand, and reported they could trust the information on the app. Those participants who had higher engagement or found the program most helpful for their sleep and their infant’s sleep showed the greatest improvement in their fatigue. Qualitative feedback from participants noted that while many found the app a lifeline with helpful information and features, others noted unintended negative impacts, such as spending too much time on their phone.

**Conclusions:**

Delivery of an infant sleep program via mHealth is effective for and acceptable to many parents. While not preferred by all parents, mHealth is an accessible, low-cost, and high-reach mode of providing information and management strategies for parents experiencing early parenting difficulties.

## Introduction

Sleep and settling difficulties during infancy and childhood are common and can be challenging for many families [1]. In an Australian study, 39% of mothers with infants at 1 month of age reported infant sleep difficulties [2]. While sleep disturbances in early infancy are normal and there is no commonly applied clinical definition of what constitutes a sleep problem in infancy [3], sleep and settling difficulties are generally understood to be related to infant regulation, with sleep difficulties potentially related to *dys*regulation [1]. Infants and their parents are interconnected in the management of infant dysregulation, as an infant needs a primary caregiver to facilitate regulation [4]. To do this, a parent needs to be attuned to their infant and to accurately observe, interpret, and respond to their infant’s cues (eg, facial expressions and crying) in a warm, consistent, and timely manner [4,5]. This skillful parenting interaction takes time to develop and can be impacted by many factors, including infant temperament, parental mood symptoms, and parents’ confidence and self-efficacy in their parenting [5].

Prevention and early support for families experiencing difficulties are important, as infant regulatory issues can continue into later infancy and toddlerhood [1,6], with significant implications for both child and parental mental and physical health. Disrupted sleep and frequent night waking in infancy and toddlerhood are associated with social, emotional, and behavioral challenges in toddlers [7] and with fatigue and mental health disorders in parents [8]. Thus, infant sleep and settling difficulties can impose a substantial burden on health systems: parents reporting infant regulatory issues often use multiple health services in their search for support [9].

Interventions providing education and/or support to parents regarding infant sleep can have positive impacts on infant sleep and maternal mood [10,11]. A Cochrane review of randomized controlled trials of postnatal education with parents of infants aged 2 months or under found interventions that included education about infant sleep led to greater length of infant sleep at 6 weeks of age [10]. A review and meta-analysis of randomized controlled trials of psychosocial and sleep-focused interventions with mothers and infants aged 12 months and under found a small but significant positive effect on infant sleep and maternal mood, as measured by the Edinburgh Postnatal Depression Scale (EPDS) [11]. A recent umbrella review of 5 systematic reviews (including 24 individual studies involving several thousand families) found that behavioral sleep interventions increased total nighttime sleep in infants and may also help reduce nighttime wakings and improve maternal mental health [12]. However, most interventions in these reviews had multiple components and were based on different approaches to infant sleep, for example, behavioral management versus intuitive parenting, making it difficult to isolate the component or approach that is most effective.

In Australia, early parenting centers such as Tresillian are a primary source of support and intervention for parents who experience challenges with their infant’s sleep [13]. Such centers have been part of the Australian health landscape for more than a century and currently provide group, day, and residential programs for parents of children aged 0 to 3 years, with challenges related to sleep, settling, feeding, and/or parenting capacity and confidence, and, in some settings, mental health support [14,15]. While these centers house considerable clinical experience and expertise, there are substantially fewer published studies reporting the efficacy of infant sleep interventions delivered by these centers, compared with behavioral sleep programs overall. However, the available evidence indicates these programs are associated with similar improvements in infant sleep and/or parent mental health to those outlined earlier [13,15-17]. A recent systematic review and meta-analysis of behavioral interventions for problematic infant sleep and unsettled behavior incorporated 16 studies, 8 of which were in Australian early parenting centers [18]. Consistent with earlier meta-analyses, this research synthesis found mild to moderate improvements in infant sleep and maternal mental health across interventions.

Most of the studies and research syntheses outlined earlier were based on face-to-face programs and consultations, and not all parents are able to attend in-person programs. Parents face a range of barriers, including long waiting lists, lack of transport, difficulty accessing childcare, and geographical isolation [19]. In addition, there has been a generational shift in the preferred sources of support sought by parents. Millennial and Gen Z parents, born from 1981 onward, who have themselves grown up in the digital age, naturally choose digital tools to support their parenting [20]. With these factors in mind, and fueled by necessity during the recent COVID-19 pandemic [21], there has been a dramatic increase in online tools and programs for parents over the past decade, to the extent that they are now ubiquitous [20]. An increasing number of these digital or mobile health (mHealth) parenting tools and programs are delivered through smartphone apps. A review published in 2020 found 421 maternal and infant health smartphone apps with approximately 25% (n=102) developed by a health care provider or affiliated provider [22].

As a problem that causes substantial anxiety for parents, infant sleep is the focus for many new parenting apps. Evidence for the efficacy and/or acceptability of these interventions is still emerging, and findings are mixed. Leichman et al [23] evaluated the real-world effectiveness of an mHealth behavioral sleep intervention among caregivers of 404 infants aged 6 to 11.9 months and found improvements in a range of sleep-related outcomes with the greatest improvements for those infants classified as problem sleepers at baseline.

While apps have been shown to be acceptable to parents for health service delivery [24,25], reviews assessing the quality of information on infant health apps indicate poor quality of information [26,27]. Apps are expensive to develop and maintain—therefore, most parenting apps are commercial enterprises, which may prioritize profit over evidence-based content [20]. Even where free evidence-based apps do exist, parents often prefer their commercial counterparts due to their superior design, interactivity, and customization [20]. Very few of the commercially available apps currently on the Google Play Store or the Apple App Store are developed by or with health organizations [22], and few have been evaluated [28]. Parenting apps take their place in an overwhelming ocean of parenting information available to modern parents. A 2022 Google search for the term “parenting” returned an astonishing 1,850,000,000 results [29], highlighting the need for high-quality information and programs that are appealing, user-friendly, readily available, and easily distinguishable from less sound material [30].

In 2019, the SleepWellBaby app was developed through a partnership between SleepFit, a sleep app development company, and Tresillian, a not-for-profit early parenting support service, in an effort to increase parents’ access to high-quality advice, education, and guidance. The content of SleepWellBaby is based on the Tresillian approach to infant sleep and settling. This approach focuses on education about normal infant or child sleep, natural sleep rhythms (eg, day or night and circadian rhythms), infant cues (communication), and responsive settling techniques such as hands-on settling [31]. Tresillian’s approach is about supporting positive parent and infant relationships through improving parents’ understanding of infant cues and supporting parents to reflect on personal or relational elements that may be impacting how they respond to their infant or child [31].

The purpose of this study was to determine the engagement with and acceptability of SleepWellBaby, an app-based parenting program to support infant sleep, and to investigate the impact of the program on parent fatigue, emotional health, and sense of competence, confidence, and self-efficacy.

## Methods

### Study Design

This was a multimethod quasi-experimental pre-post study.

### Intervention: The SleepWellBaby App

SleepWellBaby is an infant sleep app for parents. The SleepWellBaby app provides parents with a 7-day program that may be repeated as many times as users desire. It is tailored to the infant’s or child’s age and follows a feed-play-sleep daily rhythm. Parents are prompted to complete daily check-ins and sleep tracking, which help in monitoring progress over the 7-day program. There are also daily top tips, frequently asked questions, and “wisdom” cards on feeding, playing, and sleeping. The information covered includes an understanding of how normal developmental stages impact sleep, natural sleep rhythms (eg, day or night and circadian rhythms), infant cues (communication), tips for creating a safe, conducive sleep environment for infants or children, and support about infant or child settling and resettling [31]. The app also includes information and prompts for parents to access more intensive support as needed, for example, telephone support line and information on how to book a face-to-face appointment with Tresillian. Tresillian is Australia’s largest early parenting service located in New South Wales on the east coast of Australia. Tresillian provides services to thousands of families annually [32]. SleepWellBaby, while a separate program, was based on the Tresillian approach, developed with Tresillian clinicians, and is endorsed and promoted by Tresillian.

### Recruitment

Potential participants were recruited through online advertising, primarily through paid advertising on Facebook, posts on the Tresillian Facebook and Instagram social media pages, and posts on the Sydney Institute for Women, Children and their Families Twitter/X and LinkedIn social media pages. Eligible for inclusion were parents or carers who were aged 18 years and older, had infants aged less than 6 months, could read and understand the English language, lived in Australia, and had not previously used SleepWellBaby.

Potential participants followed a link to the study landing page containing participant information housed on the REDCap (Research Electronic Data Capture) platform hosted at Sydney Local Health District [33,34] and completed eligibility questions. Eligible participants completed an eConsent form, then completed the baseline survey, and received a code to access the SleepWellBaby app. Participants were recruited from April 30, 2021, to November 30, 2021. SleepWellBaby versions 1.5 to 1.7 were in use during this period.

### Measures

#### Demographic Data

At baseline, each participant reported their age, carer role (eg, mother and father), employment, education, and country of birth. They also reported information related to the family, including relationship status, number of children, main language spoken at home, and postcode. Characteristics of the infant were collected, including infant age, gender, if they were born prematurely, and current milk feeding status.

#### Engagement

Engagement with the app was measured using an Engagement Index (EI) framework [35]. The EI includes 5 subindices, which are scored between 0 and 1. These indices included click depth index, loyalty index, recency index, interaction index, and feedback index. Metrics related to each subindex were extracted from the SleepWellBaby app analytics database (detailed in Multimedia Appendix 1). To calculate an overall EI score out of 100, these subindices are summed, divided by the number of indices, and multiplied by 100. A higher score indicated higher engagement with the app. Using this framework, participant engagement in the first 7 and 30 days was calculated to generate 2 EI scores [35]. In addition, 3 self-report questions were also asked at the 7-day and 30-day surveys: parents were asked whether they were still regularly using the app (yes or no; if no, asked to further select why they no longer use the app), if they thought the program assisted in improving their sleep (program helped parent sleep scale, 5-point Likert scale, “strongly disagree” to “strongly agree”), and if the program assisted in improving their infant’s sleep (program helped infant sleep scale, 5-point Likert scale, “strongly disagree” to “strongly agree”).

#### Acceptability

Acceptability of delivering an mHealth infant sleep and settling program was measured in 2 ways. First, a Net Promoter Score (NPS) [36] was collected within the app. Second, a 10-item “Parent Satisfaction Scale” was developed based on items from previous research on parents’ engagement with an infant health app [35]. Each item was measured on a 5-point Likert scale, from 1 (“strongly disagree”) to 5 (“strongly agree”). The satisfaction scale was designed to sum into a total scale ranging from 10 to 50, with higher scores indicating higher satisfaction with the program. These items were collected at both the 7-day and 30-day follow-up surveys.

In the survey, participants were also prompted to provide text responses through open-ended questions at the 2 follow-up surveys. More than half of the participants provided open-ended text responses on their perceptions of the app. Input was requested across several open-text questions at each of the survey time points. Questions included “Please describe reasons for not using SleepWellBaby” and “Is there anything else you’d like to add about SleepWellBaby?”.

#### Parent Well-Being Outcomes

Aspects of parent well-being measured included fatigue, parenting confidence, and emotional health. These were measured at baseline, 7 days, and 30 days. Fatigue was measured via the 9-item Fatigue Severity Scale (FSS) [37]. This scale measures symptoms of fatigue, asking participants how much they agree with the items on a 7-point scale. This scale has been used in samples of mothers attending residential child and family health services [38]. A total score is calculated and ranges from 9 to 63, with higher scores indicating greater fatigue severity.

The Me as a Parent (MaaP) questionnaire was used to measure parents’ sense of competence, confidence, and self-efficacy in their parenting [39]. This 16-item scale was developed and validated in an Australian population of parents, and measures parenting self-regulation incorporating 4 domains that the authors describe as a “…global sense of competency and confidence in parenting” (p.2853) [39]. A total score is calculated and ranges from 16 to 80, with higher scores indicating a greater sense of competence, confidence, and self-efficacy in their parenting.

Emotional health was measured with 3 instruments: a short mood screener designed for the SleepWellBaby app [40], the EPDS [41], and the Generalized Anxiety Disorder 7-item scale (GAD-7) [42]. The EPDS has been commonly used in previous research on sleep programs [11], and both the EPDS and GAD-7 are widely used as screening tools for emotional and mental health. For these 2 scales, a total score is calculated and ranges from 0 to 30 for the EPDS and from 0 to 21 for the GAD-7, with higher scores indicating greater symptoms of depression and anxiety.

### Analysis

#### Engagement

Descriptive statistics, proportions, medians, means, SDs, and 95% CIs of the 7-day and 30-day EI and subindices were calculated. Parents’ continued use of the app, program helped parent sleep scale, and program helped infant sleep scale were similarly described. Program helped parent sleep scale and program helped infant sleep scale were measured with a 5-point Likert scale, with responses later collapsed into 3 levels: “strongly agree and agree,” “neutral,” and “strongly disagree and disagree.” The McNemar-Bowker test was performed to compare responses to this question at the 2 follow-up time points.

#### Acceptability

The questions related to the continued use of the app and the NPS are reported using descriptive statistics (proportions, means, and SD). Exploratory factor analysis was applied to the 10-item Parent Satisfaction Scale, which showed a 1-factor solution with good internal consistency (Kaiser-Meyer-Olkin test=0.854; Bartlett test of sphericity *P*<.001; variance of first factor, 49.355%; Cronbach α=0.88 [43]). Therefore, these 10 items were summed, producing a single “satisfaction score.” Independent 2-tailed *t* tests were used to explore differences in the NPS and satisfaction score by the following dichotomous demographic variates: education (university qualification or higher vs no university qualification), number of children (1 child only vs 2 or more children), symptoms of anxiety and depression at baseline (baseline EPDS ≥13 and/or GAD-7 ≥15 vs baseline EPDS <13 and/or GAD-7 <15), and parent perception at baseline of infant daytime and nighttime sleep problems (yes or no). Correlation between the satisfaction score and the EI was examined with Spearman ρ.

For the open-ended text responses, thematic analysis was conducted using the following Braun and Clark’s 6-step process: (1) familiarization (reading the response), (2) initial descriptive coding, (3) forming themes (by searching for patterns and comparing and contrasting codes), (4) checking themes (internally, checking that the quotes and codes sit together as a theme, and across the dataset, checking that the themes reflect the data, and checking for disconfirming data), (5) naming themes, and (6) reporting the findings [44]. Using Lumivero NVivo (version 13) software [45] (AMM used inductive coding to code all responses and then identified themes). JA and HC each read and coded a portion of the data separately. Over several meetings, discussions, and reviews, confirmation of the coding and themes was conducted by all 3 authors (HC, JA, and AMM).

#### Parent Well-Being Measures

Separate hierarchical multiple regression models were conducted to analyze the impact of engagement with the program on 4 of the outcome variables measuring fatigue (FSS), parenting confidence (MaaP), and emotional health (GAD-7 and EPDS). To ensure that outcomes were analyzed in relation to app *use* rather than the mere *download* of the app, the 7-day EI was the predictor variable for the outcomes measured at 7 days, and the 30-day EI was the predictor variable for the outcomes measured at 30 days. In addition to engagement, another predictor of interest was parents’ perception of how the app assisted in improving their own and their infant’s sleep (program helped parent sleep scale and program helped infant sleep scale). Several covariates were also included to adjust the models. The blocks were structured as follows: EI (predictor) and target outcome (dependent variable) with baseline score of target outcome (model 1); program helped parent sleep scale and program helped infant sleep scale (model 2); parent age at baseline, infant age at baseline, parent education, number of children, and pregnancy complication reported as baseline (model 3); and baseline score of the 3 other outcome variables (model 4).

IBM SPSS Statistics (version 29) [46] was used for all analyses, and *P* values <.05 were considered statistically significant.

### Ethical Considerations

Ethics approval was provided by Sydney Local Health District (RPAH Zone) Human Rights Ethics Committee (Protocol X20-0548 & 2020/ETH03215) prior to the start of the study. All study procedures were conducted in accordance with the ethical standards of the Declaration of Helsinki. Consent was obtained from all participants through an eConsent framework, including participant information and electronic consent form. Participants provided consent for research data collection and consent to access and match these data with the data collected in the SleepWellBaby app. None of the authors were involved in the development of the program.

## Results

### Participant Characteristics

**Figure 1.**
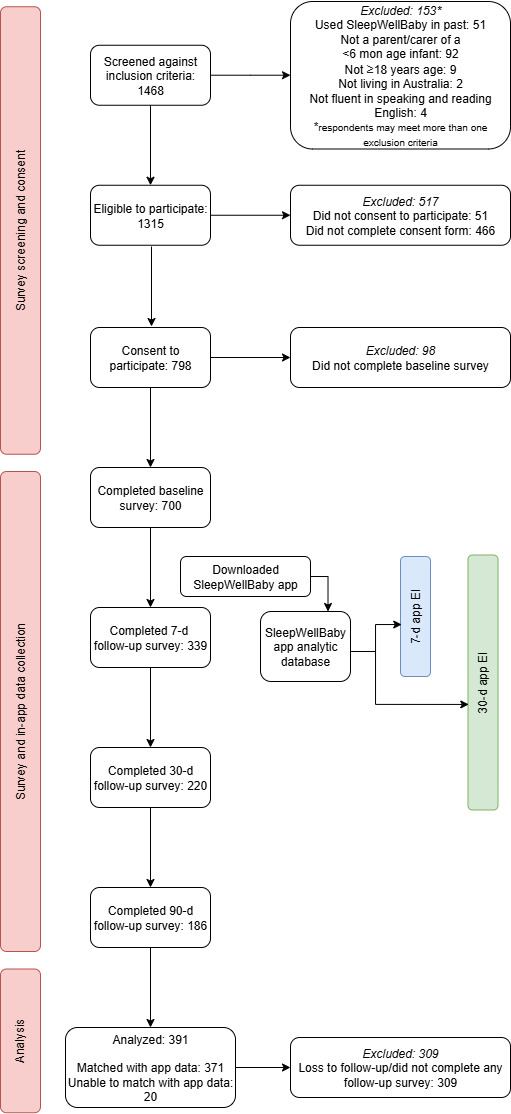
Recruitment and retention flowchart. EI: Engagement Index.

A total of 1468 potential participants indicated an interest in the app and study. Of these, 798 (54.4%) participants met eligibility criteria and consented to participate, with 700 (87.7%) participants forming the baseline sample following completion of the baseline survey. Participants in the baseline sample who did not complete either follow-up survey at 7 days or 30 days (n=309) were excluded from subsequent analysis (Figure 1).

Of the final cohort included in the 7-day or 30-day follow-up analyses (n=391), most were mothers (n=382, 97.7%), with English as their main language (n=376, 96.2%), born in Australia (n=317, 81.1%), on leave from work (n=339, 86.7%), with a university education (undergraduate: n=152, 38.9%; postgraduate: n=151, 38.6%), in a long-term or married relationship (n=381, 97.5%), with an average age of 33.2 (SD 4.01) years. More than half (n=236, 60.4%) engaged with SleepWellBaby using an Apple device, 25.8% (n=101) used an Android, and the other 13.8% (n=54) were missing these data. Most of the included infants were born at term (n=369, 94.4%), 55.5% (n=217) were male, and most were being fed breastmilk (breastfeeding: n=348, 89% and/or expressed breast milk: n=113, 28.9%), with an average age of 2.6 (SD 1.7) months at baseline and age range spanning 0 to 5.9 months. This was the first child for most participants (n=280, 71.6%). The majority of participants lived in NSW (n=269, 68.8%), followed by Queensland (n=37, 9.5%), and Victoria (n=34, 8.7%). Most participants lived in major cities (n=316, 80.8%), followed by inner regional areas (n=62, 15.9%), then outer regional areas (n=11, 2.8%), with only one participant living in a remote or very remote area (n=1, 0.3%).

There were few statistically significant differences in demographics between those lost to follow-up after the baseline survey and those who continued to complete the 7-day and/or 30-day survey (Table 1). A greater proportion of those lost to follow-up were in full-time work (5.5% vs 1.8%; *P*=.03), and fewer were breastfeeding at baseline (83.5% vs 89%; *P*=.04). Fewer participants in the low-risk category (EPDS <10) were lost to follow-up than expected by chance; however, there was no statistically significant difference between the 2 groups in the mean baseline EPDS score or the other 3 parent well-being outcomes (Table 1). We determined that participants who remained in the study for analysis were sufficiently representative on the parent well-being outcomes of the cohort initially enrolled at baseline and a complete case analysis was conducted. As may be expected, both the 7-day and 30-day EI scores were significantly higher for participants included for analysis than participants lost to follow-up (Table 1).

**Table 1.** Baseline outcomes and Engagement Index for participants and participants lost to follow-up.

Baseline outcome and engagement index	All participants at baseline (n=700)	Participants included for analysis (n=391)	Participants lost to follow-up (n=309)	*P* value
Me as a Parent Score, mean (SD)	57.26 (7.93)	57.21 (7.72)	57.33 (8.21)	.83^a^
Fatigue Severity Score, mean (SD)	40.95 (9.33)	40.89 (8.96)	41.03 (9.80)	.84^a^
Generalized Anxiety Disorder score, mean (SD)	5.20 (4.28)	4.99 (4.04)	5.46 (4.57)	.16^a^
Edinburgh Postnatal Depression Scale, mean (SD)	8.16 (4.71)	7.87 (4.60)	8.52 (4.82)	.07^a^
Probable depression (≥13), n (%)	120 (17.1)	59 (15.1)	61 (19.7)	.03^b^
Possible depression (10-12), n (%)	123 (17.6)	60 (15.4)	63 (20.4)	.03^b^
Low risk (<10), n (%)	456 (65.1)	271 (69.3)^c^	185 (59.9)^c^	.03^b^
Missing, n (%)	1 (0.1)	1 (0.3)	0 (0)	.03^b^
Positive EPDS Q10^d^, n (%)	50 (7.1)	26 (6.7)	24 (7.8)	.68^b^
Negative EPDS Q10, n (%)	650 (92.7)	365 (93.1)	285 (92.2)	.68^b^
Baby has daytime sleep problem, n (%)	540 (77.1)	306 (78.3)	234 (75.7)	.44^b^
Severity of daytime sleep problem, mean (SD)	4.73 (1.20)	4.77 (1.24)	4.69 (1.15)	.43^a^
Baby has nighttime sleep problem, n (%)	474 (67.7)	268 (68.5)	206 (66.7)	.66^b^
Severity of nighttime sleep problem, mean (SD)	4.94 (1.31)	4.91 (1.34)	4.97 (1.29)	.59^a^
Engagement Index over 7 d				<.001^a^
Mean (SD)	—^e^	73.39 (19.23)	51.81 (19.76)	
Median (IQR)	—^c^	78.29 (62.01‐88.26^f^)	52.76 (41.22-63.31^g^)	
Engagement Index over 30 d				<.001^a^
Mean (SD)	—^c^	68.52 (17.68^f^)	48.17 (17.42)	
Median (IQR)	—^c^	70.47 (57.04-82.24^f^)	47.81 (39.98-57.42^g^)	

a *t* test.

b *χ*2 test.

c Column proportions differ at the .05 level

d Question 10 of Edinburgh Postnatal Depression Scale (EPDS) asks about thoughts of self-harm.

e Not applicable.

f Participants for analysis included n=371, missing n=20 as unable to match participant with app data

g Participants lost to follow-up included *n*=264, missing *n*=45 as they did not continue with the survey or the app

### Engagement

Engagement with the program, based on the EI, was higher over the first 7 days (64.42/100), than at 30 days (60.06/100*; t*_634_=16.842; *P*<.001). While the difference is small, it indicates participants used the program more in the first 7 days, compared to the first 30 days (Table 2).

**Figure 2.**
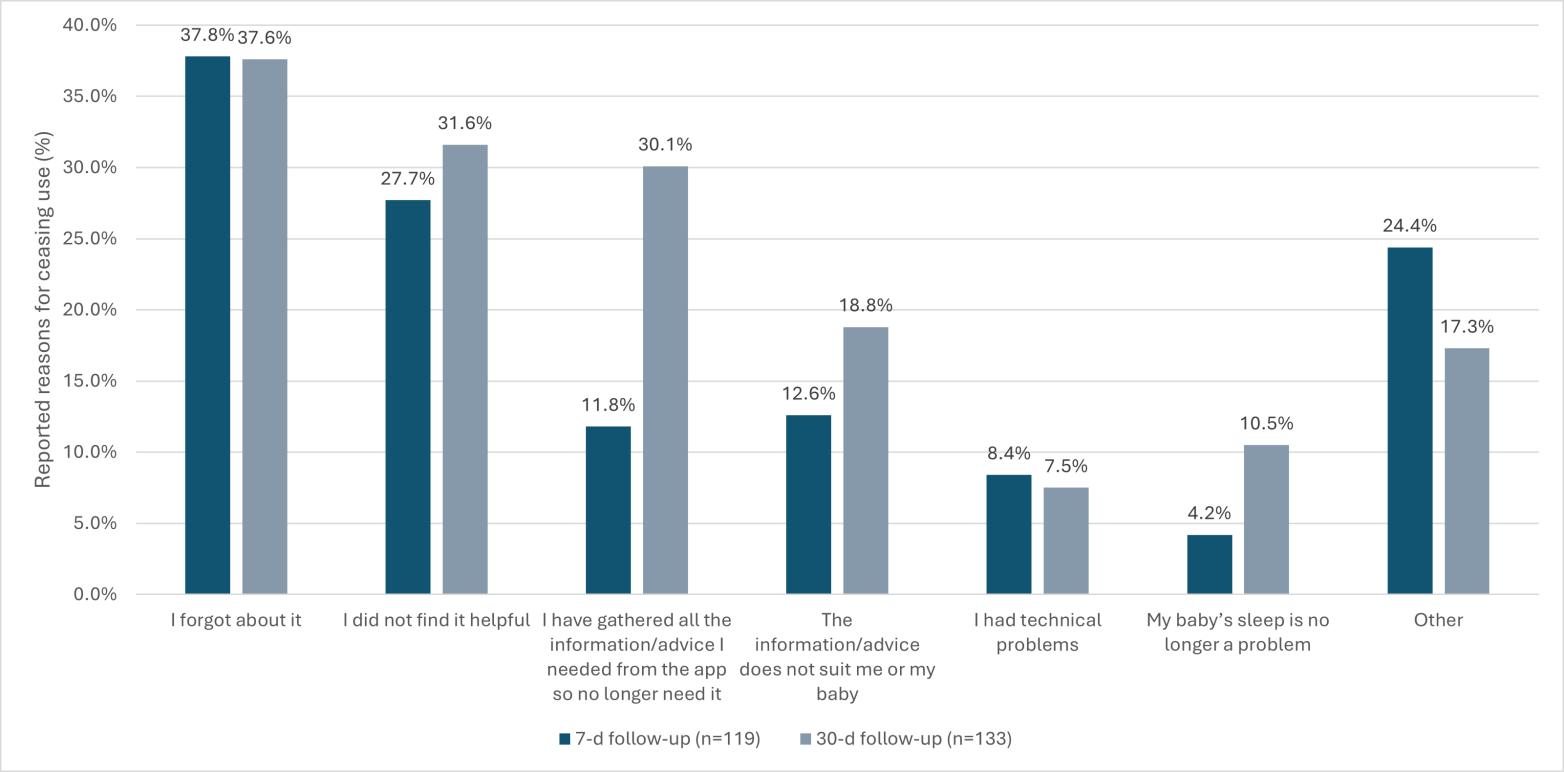
Reasons for ceasing use of the SleepWellBaby app.

Similarly, self-reported engagement based on the questions in the 7-day survey indicated decreasing use over time. At 7 days, 58.1% (n=227/391) of participants reported that they were still using the app regularly, while at 30 days, only 22.5% (n=88) were still using the app regularly. The most common reason for ceasing regular use was forgetting about the app, and the least common reason was that the infant’s sleep was no longer a problem (Figure 2). Participants could provide an open-text response explaining their choice, which was incorporated into the thematic analysis. Responses were diverse as shown in these 3 examples of the open-text response: “Too overwhelming to have to log what my baby is doing. Don’t want to continually have to look at the app P96”; “Missed a day and forgot! P127”; and It’s tricky to use P1104.”

**Table 2.** Engagement Index (EI) of app use by participants.

EI	7-d EI	30-d EI
Overall score		
Mean (SD)	64.42 (22.16)	60.06 (20.22)
Median (IQR)	65.14 (50.94‐84.20)	61.14 (45.54‐75.51)
Click depth (Ci)		
Mean (SD)	0.56 (0.26)	0.50 (0.26)
Median (IQR)	0.60 (0.40‐0.75)	0.52 (0.33‐0.69)
Loyalty (Li)		
Mean (SD)	0.80 (0.27)	0.85 (0.25)
Median (IQR)	0.90 (0.80‐0.99)	0.93 (0.86‐0.99)
Regency (Ri)		
Mean (SD)	0.87 (0.22)	0.91 (0.19)
Median (IQR)	0.95 (0.88‐0.98)	0.97 (0.92‐0.99)
Interaction (Ii)		
Mean (SD)	0.55 (0.36)	0.30 (0.33)
Median (IQR)	0.43 (0.29‐1.00)	0.13 (0.07‐0.40)
Feedback (Fi)		
Mean (SD)	0.44 (0.38)	0.44 (0.38)
Median (IQR)	0.50 (0.00‐0.80)	0.50 (0.00‐0.80)

Regarding perception of the program for help with infant sleep, most participants were either reported neutral perceptions or agreed that the program assisted with infant sleep (Table 3). There was a categorical change in perceptions between 7 days and 30 days (McNemar-Bowker test: χ²_3_=8.9; *P*=.03), with a shift from neutral to agree, indicating that more participants agreed that the program assisted with their infant’s sleep at the 30-day follow-up than at the 7-day follow-up. Similarly, on the program helped parent sleep scale, there was a categorical change in perception between 7 days and 30 days (McNemar-Bowker test: χ²_3_=8.741; *P*=.03), indicating that more participants agreed that the program assisted with their sleep at the 30-day follow-up than at the 7-day follow-up.

**Table 3.** Perception of program impact on infant and parent sleep.

Item	Disagree, n (%)	Neutral, n (%)	Agree, n (%)	McNemar-Bowker test
Did SleepWellBaby help baby sleep?				.03
7-d follow-up	61 (16.5)	201 (54.3)	108 (29.2)	
30-d follow-up	34 (15.0)	103 (45.4)	90 (39.7)	
Did SleepWellBaby help parent sleep?				.03
7-d follow-up	109 (30.1)	176 (48.6)	77 (21.3)	
30-d follow-up	64 (28.3)	91 (40.3)	71 (31.4)	

### Acceptability

For those participants who responded to the NPS question (n=306), 39.2% (n=120) rated the app 6 or less (detractors), 30.4% (n=93) rated it 7 or 8 (passives), and 30.4% (n=93) rated it 9 or 10 (promoters). The mean NPS was 7.05 (SD 2.48), indicating that most participants were satisfied with the program. However, based on the difference in the proportions of promoters and detractors, the overall NPS was −8.8% in this sample. On the other hand, the Parent Satisfaction Scale measured out of 50 showed an average of 36.26 (95% CI 35.60-36.93). The areas of language and trust scored the highest, while the areas of content and the sharing feature scored the lowest (Table 4).

There was a positive correlation between the EI and satisfaction score (Spearman ρ=0.52; *P*<.001), indicating those more highly engaged with the app reported higher satisfaction with the app. Whether engagement fostered satisfaction or whether satisfaction fostered engagement is not known. Subgroup comparisons for both the NPS and the satisfaction score found there was no difference in either scores based on baseline depression or anxiety symptoms, number of children, parent’s education, or reported infant sleep problems (Multimedia Appendix 2), indicating that a diverse range of parents with varying characteristics found the program acceptable.

**Table 4.** Parent satisfaction scale at 7-d follow-up (n=330).

Area	Question	Mean (SD)	95% CI
Overall score (range 10‐50)	—^a^	36.26 (6.17)	35.59-36.93
Language (range 1-5)	The language used in the app was easy to understand	4.25 (.623)	4.18-4.32
Trust (range 1‐5)	I can trust the information on the SleepWellBaby app	4.19 (.650)	4.12-4.26
Notifications—amount (range 1‐5)	I was happy with the number of notifications or email messages received each week	3.86 (.813)	3.77-3.95
Confidence (range 1‐5)	I felt confident using this app	3.69 (.917)	3.59-3.79
Notifications—relevance (range 1‐5)	I found the notifications or messages suited my baby’s age and stage of development	3.58 (.840)	3.49-3.67
Notifications—content (range 1‐5)	I found the notifications or email messages helpful	3.56 (.870)	3.47-3.65
Functionality (range 1‐5)	The app did everything I expected it to do	3.41 (.967)	3.31-3.51
Content (range 1‐5)	The SleepWellBaby program covered all of the things about sleep and settling that I wanted it to	3.16 (.999)	3.05-3.27
Feature—sharing (range 1‐5)	I found it helpful to share the app with my partner/family	3.14 (1.009)	3.03-3.25

a Not applicable.

### Open-Ended Text Response Findings

#### Overview

**Figure 3.**
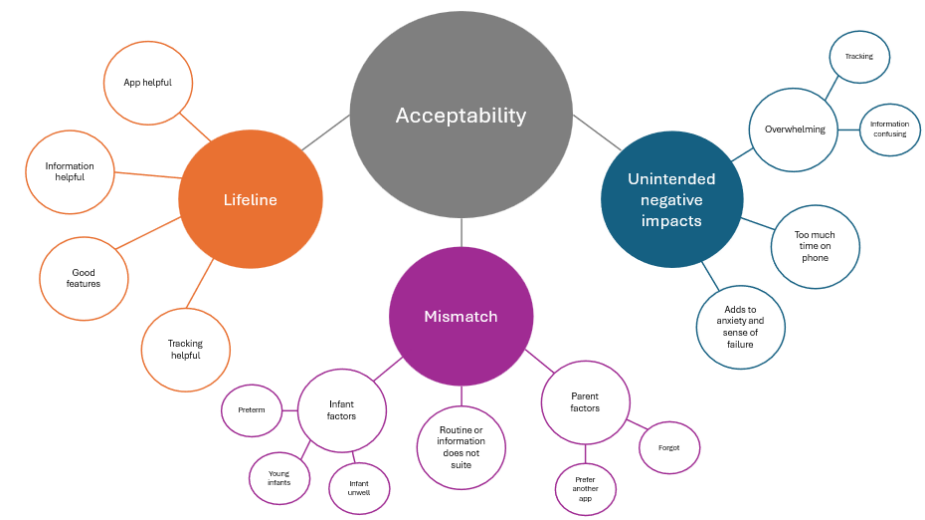
Themes on acceptability from open-ended text response.

More than half (n=226, 57.8%) of the participants provided responses to the open-ended text response fields. Three themes were identified in these responses that were relevant to the acceptability of the program. These themes and their component parts are summarized in Figure 3. The themes are described in more detail below and illustrated with direct quotations from participants. Participants are identified with their self-identified carer role, age, and infant age (noting for those infants born prematurely, this is chronological age, not corrected age). Two additional themes identified in these qualitative responses focused on challenges with using and recommendations for improving the app interface. These themes related to the earlier version of the SleepWellBaby app used in the study and are therefore not included here.

#### Theme 1: Lifeline

Responses grouped under this theme indicated satisfaction with the app and an experience of SleepWellBaby as a source of useful resources, information, and support. Participants shared that they found the program to be a helpful support for them as they navigated caring for their infant:

*This app has helped me find out the sleep rhythm of my baby and taught me to build a sleep ritual which works well at the moment*.

[Participant ID 624, mother aged 31 years with infant aged 3 months]

*So great, it felt really personal as though I had someone supporting my journey with check ins and tracking*.

[Participant ID 312, mother aged 28 years with infant aged 4 months]

*Please keep it free, parents really need this support*.

[Participant ID 215, mother aged 31 years with infant aged 2 months]

This support was noted particularly during time where access to physical services were limited during pandemic-related restrictions:

*Love it! What a wonderful program which all parents should be able to access for free – particularly in lockdown. It is the lifeline we all need in the mindless and monotonous bedlam of baby sleep while spending so much time at home*.

[P628, mother aged 33 years with infant aged <1 month]

*The program and app have taught me so much in the early newborn phase that I feel so much more confident in my ability to manage without support as lockdown continues*.

[P1085, mother aged 32 years with infant aged <1 month]

#### Theme 2: Mismatch

For some participants, however, there was a mismatch between what the program offered and their everyday experiences—they expressed that the routines or information provided did not suit. For some participants, this mismatch related to characteristics of their infant: the app was not appropriate due to factors such as prematurity, very young age, illness, or their infant’s rhythm being different from the example routines. For others, it was related to their own dispositions, such as preferring another app or forgetting about the app:

*It really didn’t suit preterm babies—this could really be worked on*.

[P168, mother aged 31 years with infant aged 3 months (chronological age)]

*...it needed the chance to adjust for preterm babies. My child was 5 weeks preemie and programs are based on my child’s actual age instead of corrected age*.

[P488, mother aged 31 years with infant aged 3 months (chronological age)]

*My baby is too young to start a routine*.

[P412, mother aged 35 years with infant aged <1 month]

*The program suggested felt a little unrealistic for my 1-month-old, and did make me feel a bit pessimistic*.

[P535, mother aged 36 years with infant aged 1 month]

*I don’t find the app helpful if my baby is off schedule.*

[P211, mother aged 24 years with infant aged 2 months]

#### Theme 3: Unintended Negative Effects

For some participants, features of the app—and, in some cases, the use of an app at all—were experienced as counterproductive, impacting negatively on their well-being, and making it more difficult to focus on being with their baby:

*Tracking everything felt overwhelming*.

[P72, mother aged 35 years with infant aged 3 months]

*I find the information and the way it is presented very confusing*.

[P532, mother aged 35 years with infant aged<1 month]

Some participants expressed that some features or use of the app added to their anxiety and sense of failure:

*I found it was making me more anxious tracking sleep etc.*

[P78, mother aged 28 years with infant aged 5 months (chronological age)]

*My baby wasn’t sleeping well and it made me feel much worse...the app made me feel I was failing*.

[P65, mother age not reported with infant aged 4 months]

Other participants also noted that it contributed to increasing time on their phone, which was seen as undesirable:

*I rarely ever was able to track in real time, [it] takes attention away from baby when they need it most*.

[P605, mother aged 32 years with infant aged 5 months]

### Impact on Parent Well-Being

#### Fatigue

Parents’ engagement and self-reported perception of the program had a significant impact on parents’ fatigue (FSS). In the final adjusted linear regression model, with all variables of interest included (model 4), 7-day engagement with the app decreased fatigue (Table 5). In this model, a 1-point increase in the 7-day EI score was associated with a decrease in FSS by 0.06 at 7-day follow-up. Similarly, the program helped parent sleep scale was also related to a decrease in FSS score, where a 1-point increase in agreement that the program helped with parent sleep was associated with a 1.7-point decrease at 7-day follow-up.

Fatigue at the 30-day follow-up was unrelated to the 30-day EI. However, in the final adjusted linear regression model (model 4), parent self-reported perception of the program was related to the FSS, where a 1-point increase in agreement that the program helped with infant sleep was associated with a 1.7-point decrease in FSS at 30-day follow-up.

**Table 5.** Impact of the engagement with app on fatigue, parenting confidence, and emotional health.^a^

Parent well-being outcome		7-d		30-d	
			*P* value		*P* value
Fatigue, adjusted FSS^b^ (95% CI)					
Engagement Index		−0.06 (−0.1 to −0.01)	.01	0.02 (−0.05 to 0.09)	.63
Program helped infant sleep		0.41 (−0.93 to 1.76)	.55	−1.74 (−3.48 to −0.004)	.05
Program helped parent sleep		−1.72 (−2.95 to −0.48)	.007	0.81 (−0.96 to 2.49)	.34
Parenting confidence, adjusted MaaP^c^ (95% CI)					
Engagement Index		0.03 (−0.01 to 0.06)	.11	0.02 (−0.04 to 0.07)	.57
Program helped infant sleep		1.48 (0.46 to 2.50)	.005	0.53 (−0.72 to 1.78)	.40
Program helped parent sleep		0.11 (−0.82 to −1.05)	.81	0.46 (−0.75 to 1.66)	.46
Emotional Health, adjusted EPDS^d^ (95% CI)					
Engagement Index		0.00 (−0.02 to 0.02)	.97	0.01 (−0.02 to 0.04)	.69
Program helped infant sleep		−0.47 (−1.07 to 0.14)	.13	0.13 (−0.57 to 0.84)	.71
Program helped parent sleep		−0.18 (−0.73 to 0.38)	.53	−0.64 (−1.13 to 0.04)	.07
Emotional Health, adjusted GAD-7^e^ (95% CI)					
Engagement Index		0.01 (−0.01 to 0.02)	.53	0.00 (−0.03 to 0.02)	.82
Program helped infant sleep		−0.34 (−0.89 to 0.20)	.22	0.15 (−0.49 to 0.79)	.64
Program helped parent sleep		−0.47 (−0.97 to 0.03)	.07	−0.48 (−1.10 to 0.14)	.13

a All models adjusted for parent age at baseline, infant age at baseline, parent education, number of children, pregnancy complication reported as baseline, baseline score of target outcome, and baseline score of the 3 other outcome variables.

b FSS: Fatigue Severity Scale.

c MaaP: Me as a Parent.

d EPDS: Edinburgh Postnatal Depression Scale.

e GAD-7: Generalized Anxiety Disorder-7.

#### Parenting Confidence

In the final adjusted linear regression model (model 4), neither the 7-day nor 30-day EI was related to the MaaP score (parenting confidence). However, the program helped parent sleep scale was related to the MaaP at the 7-day follow-up: a 1-point increase in agreement that the program helped with infant sleep was associated with a 1.48-point increase in MaaP at the 7-day follow-up. This demonstrates that participants who agree that the program is helpful for their infants’ sleep have a measured improvement in their sense of competence, confidence, and self-efficacy as a parent.

#### Emotional Health

In the final adjusted linear regression model (model 4), parents’ emotional health was not associated with engagement nor with self-reported perception of the program. Neither the EPDS nor GAD-7 scores at the 7-day or 30-day follow-up were related to the 7-day EI score or 30-day EI score (Table 5).

## Discussion

### Principal Findings

This study provides evidence of the engagement with and acceptability and impact of SleepWellBaby for Australian parents of infants. The SleepWellBaby program is suitable for many parents, and those who found the program useful experience statistically significant improvements in their fatigue and parenting confidence. Many of the participating parents engaged with the app, and the program was acceptable to most parents who provided feedback. Parents reported they were generally satisfied with the program. While acceptability and satisfaction were generally positive, there were some contrasting themes from the participants’ open-text response feedback. Importantly, it was noted that the program may at times be mismatched to parent and/or child needs and could be counterproductive with unintended negative impacts on well-being.

Nevertheless, engagement with the program was associated with a statistically significant improvement in parents’ fatigue. Parents who had higher engagement or found the program most helpful for their sleep and their infant’s sleep showed the greatest improvement in their fatigue. The modeling found that a 1-point increase (on a 100-point scale) in the 7-day EI score was associated with a decrease in parent fatigue severity (FSS) by 0.06 at 7-day follow-up. Given that a systematic review of patient-reported fatigue measures has identified minimum important difference for improvement for the FSS ranging from 0.08 to 0.4, this reduction is potentially clinically significant [47]. Similarly, parents’ self-reported perception of the program to assist their own sleep at the 7-day follow-up was associated with a 1.72 decrease in FSS, and their infant sleep at 30-day follow-up was also related to improvement in fatigue with a 1.74 decrease. While these changes are modest, it demonstrates that participants who found the program helpful, and engaged more with the program, had measured improvement in their fatigue. Regarding the other outcomes of interest, there was no indication that engagement with the program had an impact on parents’ emotional health where neither the EDPS nor GAD-7 scores at the 7-day or 30-day follow-up were related to the 7-day EI score or 30-day EI score. There was some indication that engagement with the program had an impact on sense of competence, confidence, and self-efficacy as parents’ agreement that the program helped with infant sleep was associated with a 1.48-point increase in MaaP at the 7-day follow-up.

Engagement with the program was higher for the first 7 days, compared with the first 30 days, which aligns with the design of SleepWellBaby as a 7-day program that may be repeated as needed. For those who reported discontinued use of SleepWellBaby at 7-day and 30-day follow-up, few did so when their infant’s sleep was no longer a problem. Around a third of participants had discontinued as they did not find it helpful, felt they had sufficiently gathered the information that they needed, or had simply forgotten about the app. This is similar to feedback from parents about other parenting apps [48] and suggests additional reminder features, such as push notifications, may be useful for supporting engagement. Another avenue of increasing engagement is increasing responsiveness of the app experience to user input or prompts that are based on the tracking data users enter into the app [49].

It is difficult to directly compare engagement with other similar apps due to the diversity of methods for measuring participant engagement [50]. Even among other studies using the same EI criteria, differences in the design and interactive features of each app impact the calculated EI. With this caveat in mind, compared to other studies using the EI criteria, engagement in the current study was similar or higher [24,48,51]. Likewise, the dropout of participants is similar to other evaluations of smartphone applications undertaken in the perinatal period [4,48,51,52].

While engagement and dropout in studies and evaluations of smartphone applications in the perinatal period are mixed, accessing parenting support and information via apps is increasingly popular [30]. A review of maternal and infant health smartphone applications in 2020 found 421 smartphone applications available, with 102 being ‘evidence-based apps’ as they were developed by a health care provider or affiliated provider [22]. There is a notable difference in parent use and engagement in ‘commercial apps,’ which are downloaded in the millions, and ‘evidence-based apps,’ which are downloaded in the thousands [53]. This may be due to marketing approaches and costs associated with keeping the application up to date with the smartphone operating systems. The risk in this market is that ‘commercial apps’ may not include the best, safest, most up-to-date evidence to support parents [30]. A solution may be partnership, as seen for SleepWellBaby, which combines commercial engagement strategies with evidence-based material [20].

While the NPS was negative in this sample, the 2025 12-month-to-date NPS for SleepWellBaby was 19 (email, November 24, 2025). Additionally, the adequacy of NPS as a standalone metric for use in healthcare has been questioned [54]. Therefore, we also included further questions related to satisfaction. On the basis of these, the program was acceptable to most, who were generally satisfied with the program and perceived most of the features as useful. However, some participants also expressed concerns that the program may at times be mismatched for their current needs and could be counterproductive, with unintended negative impacts on their well-being. One aspect of mismatch was the age and stage of development related to term versus premature infants. In the qualitative feedback, there was equivocal feedback regarding the suitability of the program for young infants (under 3 mo). Some parents did not find the tracking and sample plans realistic for their young infant, although others did. The Tresillian approach to infant and child sleep is child-led and responsive to child needs, and there is an emphasis on facilitating parents to observe, understand, and respond to their infant cues [31]. It may be that the nuance of this message is more difficult to convey via an app and may need additional educational resources (eg, videos).

One element within the theme *unintended negative effect* was that using digital technology made it harder for parents to focus on being with their baby. This tension around digital technology and parenting has been described elsewhere [55]. The parents in Facca’s qualitative study described the usefulness of digital technology but felt some guilt about using these devices around their young children [55]. There is emergent research identifying different styles of engagement with apps among parents, such as those who tend to use detailed, consistent tracking and those who use basic, intermittent tracking [56,57]. For SleepWellBaby, tracking is just one feature of the program, and parents can choose not to use tracking while still using other features.

While tracking of infant sleep and daily routines can be a useful tool to identify patterns and routines, qualitative feedback from some parents indicated it was counterproductive to achieving their goals. A recent cross-sectional study on breastfeeding app use found that mothers who tended to use detailed, consistent tracking, compared to those using more basic, intermittent tracking, had higher psychopathological symptoms (eg, symptoms of depression or anxiety) [56]. However, as that was a cross-sectional study, the direction of effect is not clear—ie, whether tracking within the app triggered increased mood symptoms or whether mothers who already exhibited these symptoms were more likely to undertake detailed tracking. In the current evaluation, the EI measure did include app tracking elements in the interaction index, and there was no association between the EI and anxiety symptoms. However, any app that includes tracking in the perinatal period should be developed in line with emerging evidence about any relationship between parental symptoms of depression or anxiety and app-based tracking [56].

### Strengths and Limitations

A strength of this study was an open recruitment to sample across a general population of parents with minimal exclusion criteria. Yet, while there were limited restrictions on those eligible as the sample was self-selected, the demographic of recruited parents was skewed compared to the general parenting population toward women (mothers) who spoke English as the main language at home, mostly born in Australia, on parental leave from work, university educated, married or in a long-term relationship, and residing in a major city. While reflective of the general demographic of women who access residential parenting support services [14,58], it limits the applicability of these findings to parents with broader demographic characteristics, such as parents with lower educational attainment or those living in regional and rural locations.

The findings are also limited by the quasi-experimental pre-post study design. The lack of a control group and randomization limits the ability to identify the specific impact of the program. However, to offset this limitation, a strength of our analysis is the inclusion of EI, which is an objective measure of participant engagement with the app. Including EI within the models on impact provides some support that change in participant outcomes was related to participant use of the program. The potential for selection and response bias represents an additional limitation of this study. Participants were parents and carers who were motivated to use the SleepWellBaby app and willing to share their experiences and feedback, which may limit the generalizability of the findings. Most participants perceived their infant to have a sleep problem; however, this is unsurprising given the app’s purpose of supporting parents to understand infant sleep and providing guidance on responsive sleep strategies. Although the proportion of participant loss to follow-up was comparable to that reported in other studies evaluating perinatal smartphone applications [48,52], attrition remained substantial. Participants who continued and those who did not were very similar in almost all baseline characteristics, although those participants who continued with the study were more highly engaged with the app; we therefore acknowledge the potential for overestimation of the effects of the SleepWellBaby app and limitation of generalizability of the results.

Finally, the evaluation was conducted during the COVID-19 pandemic, between April 2021 and March 2022. This period of data collection overlapped with several public health measures, including stay-at-home orders and limitations on in-person gatherings. There is growing evidence that the pandemic and associated public health measures were associated with poorer mental health outcomes for parents [59,60]. Notably, the NSW government provided funding to give free access to SleepWellBaby for NSW families during times during the pandemic when the strictest stay-at-home orders were in place (March to July 2020 and June to October 2021). Therefore, the findings need to be interpreted with this context in mind.

As noted, this sample was not particularly diverse and included mostly mothers. Few studies have included both parents [61]. One feature within SleepWellBaby is the ability to share with multiple users—however, data on the use of this feature was not collected in this evaluation. Therefore, further research could investigate the use of the program by both parents with a focus on this functionality. Importantly, any further research and evaluation should aim to recruit a diverse sample of parents to identify the efficacy and acceptability among a broader range of parents.

### Conclusions

SleepWellBaby is an infant sleep digital smartphone app that offers a useful program suitable to many parents with infants. As is the case with many interventions, it is not one-size-fits-all—not all parents will find accessing early parenting support via an app helpful. Therefore, this is a useful program to be included within a suite of support options for parents, including non–mobile phone-based interventions. SleepWellBaby is available within the early parenting support structures provided in Australia, including primary health child and family services and intensive early parenting services, such as those provided by Tresillian Family Care Centres. Within this context, SleepWellBaby offers a unique accessible support option for many parents that can promote improved parental health and well-being through reduced fatigue and increased parenting confidence.

## Supplementary material

10.2196/88948 Multimedia Appendix 1 Engagement Index equation and subindices.

10.2196/88948 Multimedia Appendix 2 Subgroup comparisons of Net Promoter Score and the satisfaction score.

## Abbreviations

EI: Engagement Index
EPDS: Edinburgh Postnatal Depression Scale
FSS: Fatigue Severity Scale
GAD-7: Generalized Anxiety Disorder seven-item scale
MaaP: Me as a Parent
NPS: Net Promoter Score
REDCap: Research Electronic Data Capture
