# Supplementary material for: Engagement, Acceptability, and Impact of a Parenting App to Support Infant Sleep and Parent Well-Being: Quasi-Experimental Study
Source: JMIR Pediatr Parent. 2026 Jun 11;9:e88948. doi: 10.2196/88948 (PMC13256176; doi:10.2196/88948)
Supplement: Multimedia Appendix 1 [file pediatrics-v9-e88948-s001.docx]

# Appendix 1

Engagement in the app was measured using an Engagement index (EI) framework. Five sub-indices make up the final EI score, which is the mean of all subindices, multiplied by one hundred. A total score is calculated and ranges from 0 - 100. A higher score indicated higher engagment with the app. Using this framework, participant engagement in the first 7 and 30 days, respectively, was calculated to generate two EI scores.

$$EI=(\frac{Ci+Li+Ri+Ii+Fi}{number of indices})\times100$$

***Click Depth Index***

This subindex is based on the pages viewed per session. A session is anytime the participant opens the app. This provides a gauge of how often participants engaged with more ‘depth’, that is, viewing multiple pages of the app. The basis of this subindex is the number of sessions having 4 or more pages viewed divided by the total number of sessions in the 7- and 30-day time period. The threshold (4 pages) was applied based on the median value of pages per session used by participants. A score of 1 would indicate that in every session the participant viewed 4 or more pages, a score of 0 would indicate a participant never viewed more than 4 pages in a session.

***Loyalty Index***

This subindex was based on the frequency of app access throughout the 7- and 30-day periods, providing a standardised score base on the number of times the participant accessed the app. The closer the score is to 1 the more times the participant accessed the app in the calculation period.

***Recency Index***

The number of days between each session formed the basis of the recency index. This sub-index was calculated based on when participants first accessed the app and the average number of days between sessions when the participant accessed the app until the 7-day period and the 30-day period, respectively. The closer the score is to 1 the more frequently the participant accessed the app in the calculation period.

***Interaction Index***

The Interaction Index was based on the number of times the participant appeared on the page or feature divided by the calculation period (either 7 or 30 days). Thirteen interactive features were included in this index:

- Daily Check-in: A short 3 question survey for parents to report their mood, sleep, and baby’s sleep
- Sleep tracking: A tracked sleep where the parent logs an period of infant sleep
- Feed tracking: A tracked feed where the parent logs an infant feeding event.
- Nappy tracking: A tracked nappy where the parent logs an infant nappy change
- Notification activated: A notification from the app has been actioned by a parent
- Wisdom card viewed: A parent has opened a wisdom card about a topic of interest
- FAQ view: A parent has tapped on an FAQ of interest to see the answer to the question
- Add planner item: The parent added a new planner item in their planner
- Edit planner item: The parent modified an existing planner item
- Play lullaby or soothing sound: A parent played a lullaby or soothing sound via the app to help assist with sleep
- Call helpline: A parent called a helpline presented in the helplines and support page
- Feed, Play or Sleep page change: A parent changed page within the program to see more detail on feed, play or sleep
- Navigate to different “timezone” in the program: A parent explored a past or upcoming “timezone” within their day of the program, e.g. during the “morning zone” 7am - 12pm the parent explored the “night zone” 7pm – 12am

To standardise this score, if the number of times the participant appeared on the interactive page or feature was greater than the calculation period, then the calculation period was taken as the denominator. Therefore, in the 7-day calculation period if a participant opened an interactive feature >7 times then they would have a score of 1. In the 30-day calculation period if a participant opened an interactive feature >30 times then they would have a score of 1.

***Feedback Index***

The net promotor score asked participants, “would you recommend SleepWellBaby to a friend” on an 11-point scale from 0 to 10. This question was asked within the SleepWellBaby app at the end of the first 7 days. This same score was used for both the 7-day and 30-day EI. This index score was divided by 10 to provide a score of 0 – 1. If a participant did not answer this question, they were coded a score of ‘0’ indicating no feedback was given.

| **Indices** | **Definition** | **Formula** | **Calculation period** |
| --- | --- | --- | --- |
| Click-Depth Index (C_i_) | The number of sessions where the number of pages viewed was ≥ 4 over the calculation period. | $\frac{\begin{aligned} Sessions having \\ "at least 4 pages viewed" \end{aligned}}{\begin{aligned} All sessions during \\ the calculation period \end{aligned}}$ | - 7 days - 30 days |
| Loyalty Index (L_i_) | How frequently participants accessed the app from commencing the program until the 7- and 30-day period | $1-\frac{1}{\begin{aligned} Number of sessions \\ accessed during the \\ calculation period \\ \end{aligned}}$ | - 7 days - 30 days |
| Recency Index (R_i_) | The time difference between each session the participant accessed the app | $\frac{1}{\begin{aligned} Average number of days \\ between visits for \\ each period \end{aligned}}$ | - 7 days - 30 days |
| Interaction Index (I_i_) | Number of times the user appears in the interaction feature | $\frac{\begin{aligned} Number of times the user \\ appears in the interaction \\ \mathrm{feature} \end{aligned}}{Calculation period}$ | - 7 days - 30 days |
| Feedback Index (F_i_) | Feedback given through subjective measure of whether the participant would recommend the SleepWellBaby app to a friend | Participant responded on a scale of 0-10:  $\frac{Participant response}{10}$ | - n/a |
