# Supplementary material for: Engagement, Acceptability, and Impact of a Parenting App to Support Infant Sleep and Parent Well-Being: Quasi-Experimental Study
Source: JMIR Pediatr Parent. 2026 Jun 11;9:e88948. doi: 10.2196/88948 (PMC13256176; doi:10.2196/88948)
Supplement: Multimedia Appendix 2 [file pediatrics-v9-e88948-s002.docx]

# Appendix 2

Subgroup comparisons of net promotor score and the satisfaction score

|  | Satisfaction score  mean (SD) | Test statistic, p-value, n | Net promotor score  mean (SD) | Test statistic, p-value, n |
| --- | --- | --- | --- | --- |
| **EPDS greater than 13 and/or GAD greater than 15 at baseline** | | | | |
| Yes n=51 | 36.31 (SD 5.70) | t = .067 (73.84), p=.95 | 6.57 (2.50) | t = 1.53 (303), p=.13, n=305 |
| No n= 278 | 36.32 (SD 6.18) |  | 7.17 (2.44) |  |
| **Number of children** | | | | |
| Only one child n=243 | 35.33 (6.35) | t= .48 (163.17) p=.63 | 6.96 (2.55) | t= -.99 (302), p=.32  n=304 |
| More than 1 child n=85 | 35.98 (5.67) |  | 7.28 (2.29) |  |
| **Education** | | | | |
| University qualification or higher n=258 | 36.56(6.05) | t = -1.61 (105.1) p=.11), n=329 | 7.03 (2.43) | t= .33 (304), p=.74, n=306 |
| No university qualification n=71 | 35.17 (6.55) |  | 7.15 (2.70) |  |
| **Daytime sleep problem at baseline** | | | | |
| Yes n=255 | 36.45 (SD 6.28) | t=-1.11 (127.46) p=.27 | 7.00 (2.47) | t=.52 (303), p=.61, n=305 |
| No n=74 | 35.58 (SD 5.77) |  | 7.18 (2.52) |  |
| **Nighttime sleep problem at baseline** | | | | |
| Yes n=227 | 36.23 (SD 6.17) | t=0.13 (196.66) p=.90 | 6.97 (2.54) | t=.95 (304), p=.34, n=306 |
| No n=103 | 36.33 (SD 6.19) |  | 7.27 (2.33) |  |
